# Exercise Reduces the Resumption of Tumor Growth and Proteolytic Pathways in the Skeletal Muscle of Mice Following Chemotherapy

**DOI:** 10.3390/cancers12113466

**Published:** 2020-11-20

**Authors:** Edson Alves de Lima, Alexandre Abilio de Souza Teixeira, Luana Amorim Biondo, Tiego Aparecido Diniz, Loreana Sanches Silveira, Dario Coletti, Silvia Busquets Rius, José Cesar Rosa Neto

**Affiliations:** 1 Immunometabolism Research Group, Department of Cell and Developmental Biology, University of São Paulo, São Paulo 05508-000, Brazil; limaea@usp.br (E.A.d.L.J.); alexandreabilio@usp.br (A.A.d.S.T.); luanabiondo@usp.br (L.A.B.); tiegodiniz@usp.br (T.A.D.); loreana.silveira@usp.br (L.S.S.); 2 Biological Adaptation and Ageing, B2A CNRS UMR 8256, INSERM ERL U1164, Sorbonne Université, 75005 Paris, France; dario.coletti@uniroma1.it; 3 Department of Anatomy, Histology, Forensic Medicine and Orthopedics, Sapienza University of Rome, 00185 Rome, Italy; 4 Cancer Research Group, Departament de Bioquímica i Molecular Biomedicine, Facultat de Biologia, Universitat de Barcelona, 08028 Barcelona, Spain; silviabusquets@ub.edu

**Keywords:** doxorubicin, chemotherapy, endurance exercise, Lewis lung carcinoma, tumor, skeletal muscle

## Abstract

**Simple Summary:**

Doxorubicin is a chemotherapeutic agent that contributes to muscle wasting. Based on the evidence that many cancer variants are associated with cachexia and that cancer patients are usually treated with chemotherapeutic agents, it is important to determine strategies to mitigate muscle atrophy. Muscle loss is a poor prognosis during cancer treatment, and exercise has emerged as a potential strategy utilized in this context. Once an ongoing regimen of chemotherapeutic treatment is not always possible, our results demonstrated that continuity of endurance exercise is a potential strategy that can be adopted when chemotherapy needs to be interrupted, minimizing the resumption of tumor growth and avoiding muscle loss.

**Abstract:**

The pathogenesis of muscle atrophy plays a central role in cancer cachexia, and chemotherapy contributes to this condition. Therefore, the present study aimed to evaluate the effects of endurance exercise on time-dependent muscle atrophy caused by doxorubicin. For this, C57 BL/6 mice were subcutaneously inoculated with Lewis lung carcinoma cells (LLC group). One week after the tumor establishment, a group of these animals initiated the doxorubicin chemotherapy alone (LLC + DOX group) or combined with endurance exercise (LLC + DOX + EXER group). One group of animals was euthanized after the chemotherapy cycle, whereas the remaining animals were euthanized one week after the last administration of doxorubicin. The practice of exercise combined with chemotherapy showed beneficial effects such as a decrease in tumor growth rate after chemotherapy interruption and amelioration of premature death due to doxorubicin toxicity. Moreover, the protein degradation levels in mice undergoing exercise returned to basal levels after chemotherapy; in contrast, the mice treated with doxorubicin alone experienced an increase in the mRNA expression levels of the proteolytic pathways in gastrocnemius muscle (*Trim63, Fbxo32, Myostatin, FoxO*). Collectively, our results suggest that endurance exercise could be utilized during and after chemotherapy for mitigating muscle atrophy promoted by doxorubicin and avoid the resumption of tumor growth.

## 1. Introduction

Cancer is a major public health concern that is responsible for significant mortality and morbidity worldwide. In 2018 it was estimated that more than 18 million people were diagnosed with this disease, and more than 9.6 million individuals succumbed to it [1]. Cachexia, one of the comorbidities related to cancer, is a multifactorial syndrome that is frequently observed in patients with cancer and is strongly associated with increased mortality [2].

Despite being an effective strategy for controlling the disease, chemotherapy frequently affects healthy cells in conjunction with cancer cells. These effects can occur concurrently with the disease treatment, or in some cases, they can have a late-onset [3]. Doxorubicin (DOX) is one of the most widely used chemotherapeutic agents in clinical practice. Regardless of its effectiveness in cancer treatment, the use of DOX should be restricted owing to its side effects, including muscle toxicity [4]. In this sense, it is important to note that muscle atrophy following chemotherapy is an independent risk factor of worsened cancer prognosis [5]. It is evident that in addition to muscle atrophy as a result of cancer itself, muscle atrophy can be an outcome of cancer treatment. This condition has negative consequences on the patient’s health. Although we know that there are several common triggers of muscle atrophy due to cancer and chemotherapy [6], the literature encompassing muscle atrophy induced by chemotherapy remains relatively unexplored [7].

Thus, the determination of molecular pathways related to cancer and/or chemotherapy is crucial, as well as the purpose of new adjuvant therapies to mitigate the muscle loss in order to induce a better antitumoral response. Pharmacological and non-pharmacological strategies have emerged in this context [8]. Physical activity is associated with a lower prevalence of various types of cancer, including colon, endometrial, breast, prostate, gastroesophageal, ovarian, pancreatic and lung cancers [9]. In fact, endurance exercise (EXER) offers an approach to managing muscle atrophy and reduces tumor growth [10,11]. Moreover, studies have suggested that in the absence of a tumor, physical exercise is able to protect the muscle during DOX treatment [12,13,14].

Over the years, there have been important advances in understanding muscle atrophy caused by cancer in conjunction with chemotherapy, as well as the development of hypotheses on how to minimize this condition [15]. Patients with cancer who maintain physical exercise during and after chemotherapy exhibit positive psychological and physical aspects [16,17]. Taking the therapeutic potential of EXER for muscle atrophy management into consideration, the aim of this study was to evaluate the effect of EXER on acute and late muscle atrophy caused by DOX therapy in an experimental model of cancer cachexia.

## 2. Results

### 2.1. EXER Performed during and after Chemotherapy Cycle Recovered Body Weight and Muscle Mass

**Figure 1:**
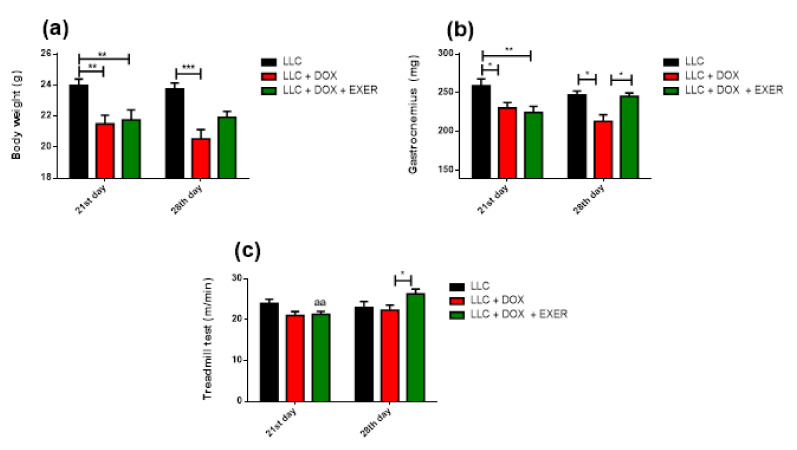
Effect of the association of endurance exercise with doxorubicin treatment on body weight gastrocnemius weight and treadmill performance immediately and delayed after the chemotherapy interruption. (**a**) Body weight (g), *n* = 9–15. (**b**) Weight of the gastrocnemius muscle (mg), *n* = 9–15. (**c**) Maximum speed on treadmill test (m/min), *n* = 9. Values represent the mean and standard error of the mean of the analysis of the data obtained. * *p* < 0.05; ** *p* < 0.01; *** *p* < 0.001; aa: *p* < 0.01 compared with the respective group on the 28th day. LLC = Lewis lung carcinoma; DOX = doxorubicin; EXER = endurance exercise.

After 21 days of tumor burden, all animals exhibited a decrease in body weight, which was further exacerbated in the mice receiving DOX with or without exercise (LLC vs. LLC + DOX and LLC + DOX + EXER, *p* < 0.01—at the 21st day). Following chemotherapy interruption, the animals of LLC + DOX group experienced a drastic weight loss compared with LLC-bearing animals (LLC vs. LLC + DOX, *p* < 0.001—on the 28th day). However, EXER performed not only during but also following chemotherapy prevented weight loss progression in tumor-bearing animals (LLC vs. LLC + DOX + EXER, *p* > 0.05—on the 28th day) (Figure 1a).

The decrease in body weight caused by DOX in the presence of a tumor is, in part, due to the decrease in both lean and fat mass. Indeed, immediately after chemotherapy (21st day), DOX decreased the weight of the gastrocnemius muscle in tumor-bearing controls (LLC vs. LLC + DOX and LLC + DOX + EXER, *p* < 0.05). During this period, EXER did not protect against the acute adverse effect caused by chemotherapy. However, after chemotherapy cycle (28th day), EXER restored gastrocnemius muscle mass (LLC + DOX vs. LLC + DOX + EXER, *p* < 0.05); in contrast, the group that remained sedentary continued to exhibit these losses (LLC vs LLC + DOX, *p* < 0.05) (Figure 1b).

### 2.2. Muscle Response to Treatments Appeared to Be Muscle-Dependent

Treatments did not affect extensor digitorum longus (EDL) weight. This muscle was only influenced by time in the presence of the tumor. There was a difference in EDL weight between the 21st and 28th days between all groups (21st day vs. 28th day, *p* < 0.05) (Table S2). Only the acute toxic effects of chemotherapy, rather than the late-onset effects, were responsible for the decrease in soleus muscle weight (LLC vs. LLC + DOX and LLC vs. LLC + DOX + EXER, *p* < 0.05, 21st day). When chemotherapy was suspended (28th day), the weight of this tissue returned to the level detected in tumor-bearing mice (LLC) (Table S2).

There was no change in the weight of the tibialis anterior muscle immediately after chemotherapy (21st day, *p* > 0.05). The group of animals that continued to exercise after chemotherapy exhibited tibialis anterior muscles with greater masses compared with the group that received only chemotherapy (LLC + DOX vs. LLC + DOX + EXER, *p* < 0.05, 28th day) (Table S2).

Immediately after chemotherapy, both groups that had been treated with DOX showed a decrease in adiposity index (LLC vs. LLC + DOX and LLC vs. LLC + DOX + EXER, *p* < 0.05, 21st day). The presence of the tumor for an additional week reduced the fat mass across all groups (21st vs. 28th day, *p* < 0.05). This decrease, however, was exacerbated in the group that was treated with only DOX (LLC vs. LLC + DOX, *p* < 0.01, 28th day); therefore, the continuity of exercise throughout the chemotherapy interruption period prevented a more precipitous drop in the weight of these adipose cushions (LLC vs. LLC + DOX + EXER, *p* > 0.05, 28th day) (Table S2).

### 2.3. Adaptive Response to Exercise Remained after Chemotherapy

Immediately after chemotherapy (21st day), the treadmill performance between the groups was similar (*p* > 0.05). However, after chemotherapy, the physical performance on the treadmill test was increased in the group of animals that continued to exercise (LLC + DOX + EXER, *p* < 0.01, 21st vs. 28th day). In contrast, those who received only chemotherapy and remained sedentary (LLC + DOX) showed worse performance compared with animals who continued to exercise (LLC + DOX + EXER) after chemotherapy (LLC + DOX vs. LLC + DOX + EXER, *p* < 0.05, 28th day) (Figure 1c).

### 2.4. Exercise Prevented the Resumption of Tumor Growth after Chemotherapy

**Figure 2:**
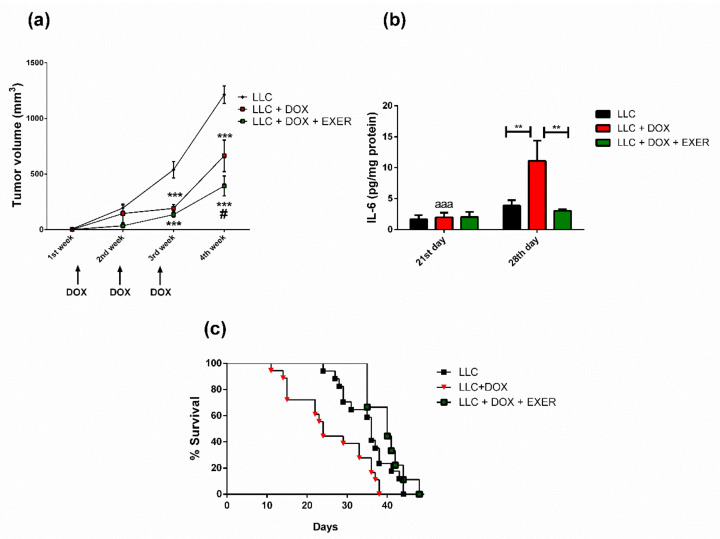
Effect of chemotherapy treatment and of the combination of endurance exercise on tumor growth. (**a**) Tumor growth curve (mm^3^). *n* = 20–18. *** *p* < 0.001 vs. LLC group. # *p* < 0.05 vs. LLC + DOX group. (**b**) IL-6 concentration (pg/mg protein) in tumor homogenate. *n* = 5–6. ** *p* < 0.01; aaa: *p* < 0.001 compared with the respective group on the 28th day. (**c**) Effect of doxorubicin and endurance exercise on the survival of mice. The survival was assessed using the Kaplan–Meier curve and was compared using a log-rank test for trend. *n* = 18–9. Values represent the mean and standard error of the mean of the analysis of the data obtained. LLC = Lewis lung carcinoma; DOX = doxorubicin; EXER = endurance exercise.

As expected, DOX was effective in delaying tumor growth. The mice in which DOX was administered exhibited a decrease in tumor growth rate immediately after chemotherapy (*p* < 0.001). However, in the period after chemotherapy (28th day), mice that received DOX and remained sedentary showed an increased rate of tumor growth in a higher proportion than those that had continued to exercise (LLC + DOX vs. LLC + DOX + EXER, *p* < 0.05) (Figure 2a). During the period after chemotherapy, IL-6 expression increased in tumors from the group treated only with DOX; however, EXER reverted this effect (LLC + DOX vs. LLC and LLC + DOX + EXER, *p* < 0.01) (Figure 2b).

### 2.5. EXER Improved Survival Rate Reduced by DOX

Tumor-bearing mice treated with DOX therapy showed a decreased survival rate compared with those that were not undergoing chemotherapy (LLC vs. LLC + DOX, *p* < 0.001). Tumor-bearing mice (LLC) had a median survival length of 36 days; with doxorubicin administration (LLC + DOX), this interval was reduced about 8 days. However, when chemotherapy was combined with EXER, we observed mitigation of the deleterious effect of chemotherapy (LLC + DOX vs. LLC + DOX + EXER, *p* < 0.001) (Figure 2c).

### 2.6. Catabolic Markers in Gastrocnemius Muscle Were Reduced with the Maintenance of Physical Exercise

**Figure 3:**
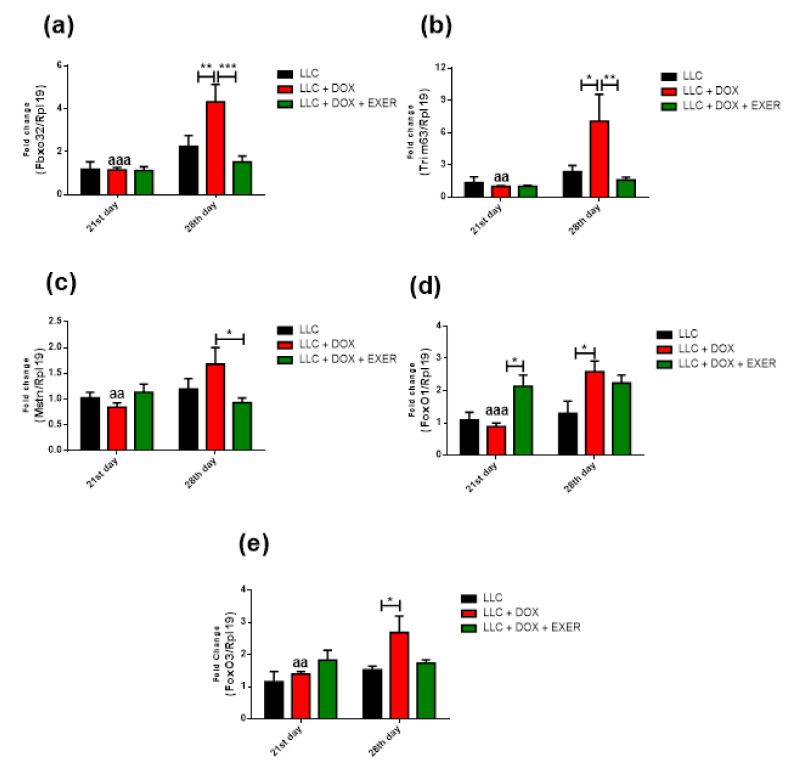
Gene expression of proteins involved in the activation of muscle catabolism. In gastrocnemius muscle, treatment with doxorubicin stimulated the atrophic pathways, while the combination with resistance exercise prevented this effect. (**a**) *Fbxo32* gene expression; (**b**) *Trim63* gene expression; (**c**) myostatin gene expression; (**d**) *FoxO1* gene expression; (**e**) *FoxO3* gene expression. Values represent the mean and standard error of the mean of the analysis of the data obtained *n* = 5–6. * *p* < 0.05; ** *p* < 0.01; *** *p* < 0.001; aa: *p* < 0.01 compared with the respective group on the 28th day. aaa: *p* < 0.001 compared with the respective group on the 28th day. LLC = Lewis lung carcinoma; DOX = doxorubicin; EXER = endurance exercise.

**Figure 4:**
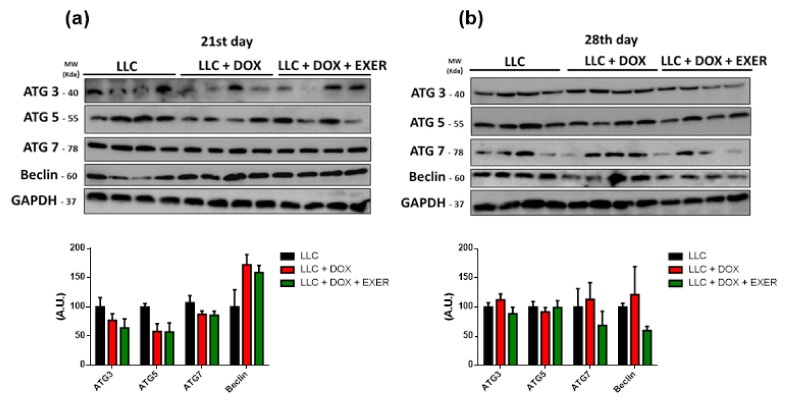
Autophagic signaling in gastrocnemius. (**a**) Expression of proteins involved in autophagic activation at the end of the chemotherapy cycle. (**b**) Expression of proteins involved in autophagic activation after chemotherapy interruption. Values represent the mean and standard error of the mean of the analysis of the data obtained *n* = 4. LLC = Lewis lung carcinoma; DOX = doxorubicin; EXER = endurance exercise. The uncropped Western Blots are available in Figure S1.

As a consequence of DOX therapy, the activation of signaling pathways associated with atrophy in skeletal muscle mostly increased in the period after chemotherapy suspension (LLC + DOX) (Figure 3). Chemotherapy contributed to the increase in *Fbxo32* expression (LLC vs. LLC + DOX, *p* < 0.01, 28th day). However, EXER during and after chemotherapy was able to prevent this enhancement (LLC + DOX vs. LLC + DOX + EXER, *p* < 0.001, 28th day) (Figure 3a). There was a late-onset increase in *Trim63* expression (21st vs. 28th day, *p* < 0.01) in sedentary animals treated with DOX (LLC + DOX). Although they also received chemotherapy, exercised animals were protected against this response and did not show increased *Trim63* expression (LLC vs. LLC + DOX; LLC + DOX vs. LLC + DOX + EXER, *p* < 0.05, 28th day) (Figure 3b). DOX produces late-onset adverse effects; this chemotherapeutic agent increased myostatin gene expression between the 21st and 28th day (*p* < 0.01). This increment, however, was mitigated by EXER (LLC + DOX vs. LLC + DOX + EXER, *p* < 0.05, 28th day) (Figure 3c). The LLC + DOX group showed an increase in *FoxO1* gene expression on the 28th day (LLC vs. LLC + DOX, *p* < 0.05). Exercised mice showed an elevated *FoxO1* gene expression on the 21st day (LLC + DOX vs. LLC + DOX + EXER, *p* < 0.05); however, on day 28, the levels did not differ from those in other groups (*p* < 0.05) (Figure 3d). One week after chemotherapy (28th day), the group of mice that had received DOX (LLC + DOX) showed an increase in *FoxO3* expression; EXER was able to prevent it (*p* < 0.05, 28th day) (Figure 3e). There were no changes in the expression of autophagy markers Beclin, Atg3, Atg5 or Atg7 immediately nor one week after the last dose of DOX (*p* < 0.05) (Figure 4).

### 2.7. DOX Contributed to the Decrease in Protein Synthesis Even One Week after Chemotherapy Discontinuation

**Figure 5:**
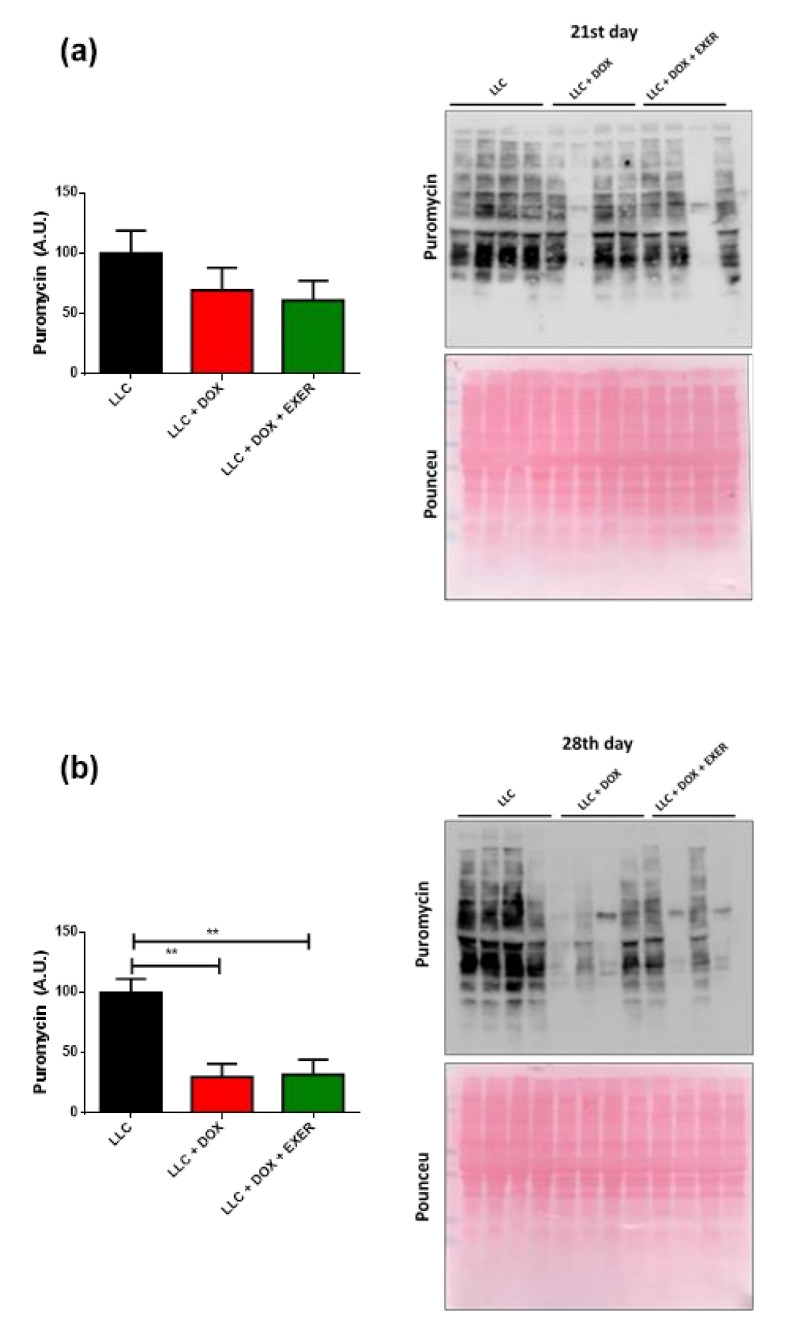
Evaluation of total protein synthesis immediately after and one week post-chemotherapy. (**a**) Total protein synthesis in the gastrocnemius muscle evaluated by the SUnSET assay at the end of the chemotherapy cycle; (**b**) total protein synthesis in the gastrocnemius muscle evaluated by the SUnSET assay after the suspension of chemotherapy. Values represent the mean and standard error of the mean of the analysis of the data obtained. *n* = 4. ** *p* < 0.01. LLC = Lewis lung carcinoma; DOX = doxorubicin; EXER = endurance exercise. The uncropped Western Blots are available in Figure S2.

There was no change in total protein synthesis immediately after chemotherapy (21st day) (Figure 5a). However, we observed a late-onset response caused by chemotherapy one week after the discontinuation of DOX therapy (28th day). There was a decrease in total protein synthesis in animals that had received chemotherapy (LLC vs. LLC + DOX, *p* < 0.01); EXER did not prevent this response (LLC vs. LLC + DOX + EXER, *p* < 0.01) (Figure 5b).

### 2.8. Expression Levels of Cytokines and Proteins Involved in Inflammatory Signaling in the Gastrocnemius Muscle

**Figure 6:**
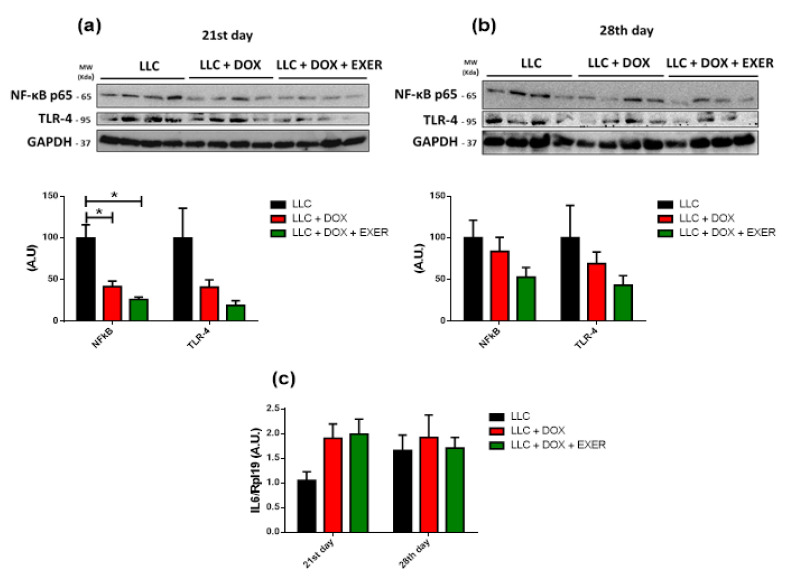
Inflammatory signaling in skeletal muscle. (**a**) Protein expression of TLR-4 and NF-κB in the gastrocnemius muscle immediately after chemotherapy. *n* = 4. (**b**) Protein expression of TLR-4 and NF-κB in the gastrocnemius muscle in the post-chemotherapy period. *n* = 4. (**c**) Gastrocnemius *IL-6* gene expression. *n* = 5–6. Values represent the mean and standard error of the mean of the analysis of the data obtained. * *p* < 0.05. LLC = Lewis lung carcinoma; DOX = doxorubicin; EXER = endurance exercise. The uncropped Western Blots are available in Figure S3.

We determined the expressions of NF-κB and TLR-4 in the gastrocnemius muscle, as well as the levels of pro- and anti-inflammatory cytokines in the muscle homogenate. Immediately after chemotherapy (21st day), there was a decrease in NF-κB p65 phosphorylation in animals treated with DOX (LLC vs. LLC + DOX and LLC + DOX + EXER, *p* < 0.05), which did not manifest the same way during the following week (*p* > 0.05, 28th day). In contrast, TLR-4 expression did not change during the experimental protocol, although there was a tendency toward a decrease in the groups treated with DOX (LLC + DOX and LLC + DOX + EXER) (Figure 6a,b). There was no change in the levels of IL-1β, IL-1 ra, TNF-α and IL-10 in the period immediately after chemotherapy (21st day) (Table S3). Furthermore, *IL-6* gene expression in the gastrocnemius muscle did not change with the treatments (Figure 6c).

### 2.9. Serum Parameters

Compared with the group that received chemotherapy alone, the exercised group had lower serum lactate levels in the period immediately after chemotherapy (LLC + DOX vs. LLC + DOX + EXER, *p* < 0.05, 21st day), although this decrease was not statistically significant compared with the group that did not receive the drug (LLC vs. LLC + DOX + EXER, *p* > 0.05). This did not prevent the delayed increase in serum lactate levels (21st vs. 28th day, *p* < 0.05) resulted from DOX therapy (LLC vs. LLC + DOX and LLC + DOX + EXER, *p* < 0.05, 28th day) (Table S4). Total cholesterol and triglyceride levels did not change during the experimental protocol (Table S4).

### 2.10. Glucose Metabolism in Tumor-Bearing Mice Undergoing Chemotherapy

Although we observed time-based effects during tumor monitoring (21st vs. 28th day, *p* < 0.05), we did not observe differences in glucose and insulin levels across different protocols (DOX or DOX + EXER) (Table S5). The fasting glucose level in mice treated only with DOX (LLC + DOX) was similar between the 21st and 28th day. In contrast, the animals that did not receive therapeutic resources (LLC) demonstrated an increase in serum glucose levels during this period (*p* < 0.01). A similar response was observed in animals that that were subjected to EXER and chemotherapy (LLC + DOX + EXER, *p* < 0.01). DOX did not alter glucose tolerance and insulin sensitivity in relation to the LLC group (Figure S4).

## 3. Discussion

Because of chemotherapy side effects, completing the full duration of a cancer patient’s treatment is not always feasible. Although the beneficial effects of exercise in cancer patients have now been established, further data from preclinical models are necessary to effectively assess exercise training protocols in the presence of cancer cachexia and chemotherapy. First, our findings showed that EXER is safe in tumor-bearing mice. Second, the combination of EXER and chemotherapy administration did not impair the antitumor effects of this anthracycline. Most importantly, exercise reduced the resumption of tumor growth after the interruption of pharmacological treatment and extended the survival of mice previously treated with DOX. Additionally, EXER was able to reduce muscle atrophy, which significantly affects on patient’s prognosis, survival and quality of life. Taken together, our results demonstrate that EXER is an efficient and effective strategy to manage the hallmark skeletal muscle atrophy caused by DOX in the period after chemotherapy. This effect can be attributed, in part, to the decreased expression of proteins related to muscle catabolism.

It has already been demonstrated that EXER offers a plethora of defenses against the growth and development of tumors [9]. However, few studies have shown the effect of EXER as a co-treatment in the presence of chemotherapy. In an interesting study, Martin-Ruiz et al. 2020 showed that additive effects of combined exercise training (sessions of endurance training and strength training) as a synergistic strategy with immunotherapy against non–small cell lung cancer [18].

Exercise can serve as a great tool for the management of chemotherapy side effects. Although DOX is a broadly used therapeutic resource in the treatment of patients with cancer, its use must be restricted due to its adverse side effects [19]. In many organs, these side effects may even manifest straight after the end of the chemotherapy cycle. This tension between an incredibly versatile treatment tool and its detrimental side effects presents the perfect environment for an adjuvant intervention such as exercise.

The combination of EXER with a short chemotherapy cycle alleviated weight loss and delayed muscle atrophy caused by chemotherapy. This protection, in turn, is due to the minimization of muscle catabolism caused by DOX, particularly with that linked to the activation of the ubiquitin-proteasome system. The use of DOX in oncological conditions is widely described in the literature and is therefore expected to lead to reduced tumor growth, particularly in solid tumors [20], such as LLC. However, decreased tumor growth rate alone does not guarantee protection against muscle atrophy, which demonstrates that in this context, pharmacological treatment has direct relevance. Moreover, DOX therapy reduces cardiovascular and muscular performance, and these parameters are very relevant to the overall quality of life of surviving patients; the utilization of EXER prevented the worsening of these key metrics [21,22].

Our findings demonstrate the role of EXER during and after chemotherapy in the management of late-onset muscle atrophy caused by this common pharmacological therapy. The myotoxicity associated with chemotherapy can persist past the end of treatment [23]. We know that the toxic effect of DOX on cardiac tissue can occur years after the end of treatment [24], and this effect is poorly documented for other tissues. The increase in muscle catabolism even after chemotherapy suspension demonstrates the relevance of our findings. DOX therapy did not completely impair the adaptive response to EXER; this adaptive response served to ameliorate a portion of the negative effects of chemotherapy.

Over the past few years, the contribution of chemotherapeutic agents to muscle atrophy has been increasingly discussed. This is important because muscle atrophy is a well-known and recurrent outcome for patients with cancer undergoing this kind of treatment. The increased degradation of sarcomeric proteins obtained via the activation of the ubiquitin-proteasome system is one of the factors that contribute to muscle atrophy. Atrogin1 and MuRF1 are ubiquitin E3-ligases selectively expressed in skeletal muscles, which are important for the progression of muscle atrophy through their promotion of protein polyubiquitination prior to 26 S proteasome targeting [25]. While ubiquitination is an essential step for protein degradation in this system, the transcription of Atrogin1 and MuRF1 are commonly used as key markers of activation. Atrogin1 and MuRF1 increased during muscle atrophy, and, as such, it is verified with the use of DOX [12]. On the other hand, endurance and resistance exercise protects against the disorders in the skeletal muscle resulted from the use of doxorubicin therapy [11,13,26]. In this study, we demonstrated that DOX upregulates the expression of these proteins and that EXER performed during and after chemotherapy minimizes muscle atrophy caused by this pharmacological treatment. This effect is partly related to the inhibition of increased ubiquitin E3-ligase expression after chemotherapy.

The transcriptional regulation of atrogenes comes from many pathways, including Akt/FoxO, (IκBα)/nuclear factor kappa B (NFκB) and MAPKs [27]. The Akt/FoxO pathway plays an important role in proteolysis due to the fact that its signaling leads to the transcriptional increase in both ubiquitin ligases (*Fbxo32* and *Trim63*) [27]. FoxO 1/3 transcription increases under atrophic conditions and is a highly regulated process [28]. DOX can decrease Akt phosphorylation in soleus muscle [29]; this decrease in phosphorylation favors the translocation of FoxO 1/3 to the nucleus, thereby leading to the expression of ubiquitin ligases. In this study, we demonstrated that transient and short-term treatments with DOX increase FoxO 1/3 transcription during the period post-chemotherapy. However, when combined with EXER, this effect was attenuated.

Another possible contributing factor to muscle atrophy caused by DOX therapy was the increase in myostatin expression. The upregulation of myostatin/SMAD signaling is one of the main negative regulators of muscle growth [30]. The increased expression of this protein is observed during muscle atrophy and has also been identified as a target for pharmacological inhibitors in an attempt to preserve lean mass during DOX therapy [4]. Therefore, the decrease in myostatin expression caused by physical exercise found in our model seems to be crucial in combating muscle atrophy. This effect has been reported both in endurance and resistance exercises [31,32]. Furthermore, even a short period of EXER is able to prevent an increase in myostatin expression caused by DOX [12]. The myostatin gene promoter is classically activated by FoxO1 and SMAD proteins [33], and the increase in glucocorticoids also contributes to this mechanism by activating the glucocorticoid responsive element [34]. Collectively, these data suggest that the decrease in myostatin expression by EXER is partly due to the decrease in corticosterone levels [21] and attenuation of FoxO signaling. In addition to reducing protein synthesis, the signal transduction of myostatin binding at its receptor provides an increase in proteolysis mediated by the ubiquitin–proteasome system [35]. Therefore, the repression of atrogenes transcription promoted by EXER after chemotherapy is also partly due to the decrease in myostatin expression.

The development and progression of cancer are constantly associated with an increased subclinical inflammatory state [36]. Physical exercise, on the other hand, has a systemic anti-inflammatory action, particularly when it is performed at moderate intensity [37]. In this sense, some of the beneficial effects of physical exercise as an adjuvant to cancer treatment come from immune and inflammatory changes [37]. To investigate the possible factors that contribute to delayed muscle mass loss caused by DOX, we investigated changes in cytokine expression and NF-κB phosphorylation. In cancer, pro-inflammatory cytokines play important roles in intensifying muscle atrophy. Many of them lead to an increase in NF-κB signaling resulting in intensified protein catabolism [38]. This is one of the mechanisms that favor protein catabolism via proteasomal degradation. However, in our experimental model, DOX therapy led to a decrease in NF-κB phosphorylation, and exercise did not alter the activation of this transcription factor.

The production of inflammatory factors during cancer by tumor cells and the host triggers muscle atrophy. IL-6 is one of the main cytokines responsible for regulating skeletal muscle homeostasis, in which the sustained increase in its levels negatively affects protein balance. This leads to muscle atrophy and the progression of cancer cachexia [39]. Although many cell types produce IL-6, evidence suggests that the tumor microenvironment is an important source of this cytokine, which leads to increased systemic concentrations. The effect is so pronounced that in animal models when the primary tumors are surgically extracted, a decrease in IL-6 levels and mitigation of the cachectic state are observed [40]. A similar effect occurs when a cytokine antibody treatment is used [41]. LLC tumors that constitutively express a high level of IL-6 develop significant cachexia [41]. In addition, DOX increases circulating IL-6 levels [42]; however, physical exercise can reduce the transcription of IL-6 in the tumor [43]. Indeed, in our experimental system, chemotherapy promoted an increase in tumor IL-6 expression, which was mitigated by the incorporation of EXER. Muscle catabolism is caused by IL-6-mediated activation of the IL-6/gp130/Stat3 pathway [44]. In particular, gp130 signaling, which is important for FoxO3 activation during cachexia promoted by LLC cells [45] and blocking Stat3 signaling, which is able to reduce muscle atrophy [46]. Additionally, when LLC cells that express high amounts of IL-6 are used as an experimental cancer model, there is a reduction in survival [47]. In turn, physical exercise has been used as a non-pharmacological approach during cancer, regulating the symptoms of the disease, the treatment side-effects and protecting against a condition of disability or premature death [48].

Autophagy is an important catabolic process involving the degradation of old and malfunctioning cellular components [49]. An increasing number of studies have demonstrated the importance of autophagy during muscle atrophy. The loss of homeostasis caused by high levels of autophagic flow is related to several pathologies [49], in which an imbalance in the fine control of autophagy levels in skeletal muscle favors muscle atrophy and the development of metabolic disturbs [50]. DOX therapy increases the expression of the regulatory proteins responsible for autophagy, whereas EXER protects against this effect [51]. However, in the presence of a tumor, despite the importance of autophagy in muscle atrophy, our results did not find an intensification of the autophagic flow when using DOX therapy in the LLC model.

On the other side of this balance in the maintenance of protein turnover, there is the ratio of protein synthesis. Studies have shown that DOX therapy decreases the ratio of protein synthesis [4,21]. Animals treated with DOX showed a decrease in the expression of a pivotal pathway, the insulin-AKT-mTOR, which is the central player of protein synthesis [52]. Our results showed that the decrease in protein synthesis significantly changes one week after the end of the chemotherapy cycle; EXER was not able to mitigate this reduction.

Fatigue is another frequently reported side effect during chemotherapy, which has been associated with worsened quality of life. Many factors explain chronic fatigue: (i) decrease in respiratory muscle mass in the diaphragm [53], (ii) cardiotoxicity [22], and (iii) decrease in skeletal muscle mass [11,21]. DOX can impair skeletal muscle function in many different ways, which largely explains the fatigue reported by patients receiving chemotherapy. This drug impairs the neuromuscular junction [54] and increases the mitochondrial damage [55]. To minimize the burden of cachexia as well as to manage this fatigue condition, EXER has been prescribed as a non-pharmacological strategy [8,56]. This type of intervention has shown desirable outcomes with improvements in mitochondrial functionality [57] and the preservation of neuromuscular junctions [54].

Therefore, it is noteworthy that endurance exercise is a safe strategy to improve muscle atrophy during and after chemotherapy treatment, probably through the inhibition of the ubiquitin–proteasome pathway. Furthermore, exercise post-treatment reduced tumor volume and the pro-inflammatory cytokine IL-6 in addition to restoring muscle mass. This study denotes not only the importance of physical activity during cancer treatment but also the crucial role of exercise continuity, especially after chemotherapy, in restoring/maintaining patients’ quality of life.

## 4. Materials and Methods

### 4.1. Animals

C57 BL/6 mice (8–10 weeks old) were used in this study. The animals were kept in a 12–12 h light–dark cycle room maintained at a temperature of 23 °C ± 2 °C, ad libitum access to food (Nuvital food from Nuvilab, Colombo, PR, Brazil) and water during the treatment. All procedures in this study followed the ethical principles of animal experimentation and were approved by the Ethics Committee on Animal Experimentation of the University of São Paulo (CEUA 96/2015) on 25 August 2015.

### 4.2. Experimental Design

On day 0, all mice were subcutaneously inoculated with Lewis lung carcinoma (LLC) cells into the right flank. After 7 days, the tumor was established. We divided the mice into three subgroups: LLC without treatment (LLC); LLC injected with intraperitoneal DOX twice a week (2.5 mg/kg body weight) until the concentration reached 10 mg/kg body weight (LLC + DOX); and DOX therapy and moderate EXER (60% maximal speed test, 5 days per week) on a treadmill (LLC + DOX + EXER). The LLC group was injected with the same volume of saline (0.9%). Each subgroup underwent two different protocol lengths: 21 days (euthanasia right after last chemotherapy dose) or 28 days (euthanasia one week after last chemotherapy dose). The LLC + DOX + EXER group ran for an extra week in the absence of chemotherapy (after 21 days) and thus, completed the experiment after 28 days. The same groups were used for determining the experimental survival rate.

### 4.3. Subcutaneous Implantation of Tumor Cells

LLC were maintained in Dulbecco’s modified Eagle medium (GIBCO, Invitrogen, NY, USA) supplemented with penicillin (100 U/mL), streptomycin (100 μg/mL), and 10% fetal bovine serum (Atlanta Biological, Lawrenceville, GA, USA). They were cultured and maintained in a tissue culture flask at 37 °C in a humidified atmosphere containing 5% CO_2_. A total of 5 × 10^5^ viable cells were subcutaneously injected into the right flank of the mice (diluted with 0.9% saline). Cell viability was assessed using trypan blue solution (Thermo Fisher, Carlsbad, CA, USA).

### 4.4. Body Composition and Tumor Volume

Tumor volume was monitored throughout the treatment and was determined using a pachymeter. Tumor length (a), width (b) and height (c) were used to determine tumor volume (mm^3^) = abc/2 [58]. After euthanasia, the weight of the soleus, gastrocnemius, extensor digitorum longus (EDL), tibialis and retroperitoneal, epididymal, mesenteric and subcutaneous adipose cushions were measured. The adiposity index was calculated as the sum of these adipose cushions.

### 4.5. Exercise Protocol

EXER started one week after tumor inoculation. EXER was performed for 2 weeks (for the 21-day protocol) or for 3 weeks of training (for the 28-day protocol) and comprised of five sessions per week, with each session lasting 40–60 min. The training speed on the treadmill was moderate and was set at 60% of the maximum speed of the mice. To determine the maximum speed on the treadmill, an incremental speed test was performed, which began with warm-up at 10 m/min. The speed was increased by 3 m/min every minute until the animals were exhausted. This assessment was initially conducted in the period prior to the experimental protocols in order to determine the initial workload, and during the last week in order to assess changes in the running performance of the mice on the treadmill. All groups were submitted to this evaluation.

### 4.6. Sample Collection

Mice were subjected to 6 h of food restriction and then euthanized for tissue and blood collection. Skeletal muscles, adipose tissues and tumors were weighed and stored at −80 °C for further analysis.

### 4.7. Evaluation of Serum Parameters

Glycemia, lactate, triglycerides and total cholesterol levels were evaluated in serum samples using a colorimetric test (Labtest kit, Lagoa Santa, MG, Brazil, Ref. 133, 138, 87 and 76, respectively). Insulin was quantified using an immunoassay technique (enzyme-linked immunosorbent assay). The insulin kit was obtained from Millipore Corp. (Bedford, MA, USA, Ref. EZRMI-13 K). The tests were performed on an EON spectrophotometer (BioTek Instruments, Inc., Winooski, VT, USA) with the ideal optical density established in the manufacturing manual of each kit.

### 4.8. Cytokine Expression

The gastrocnemius muscle and tumor was homogenized in RIPA buffer (0.625% Nonidet p-40, 0.625% sodium deoxycholate, 6.25 mM sodium phosphate and 1 mM ethylenediaminetetraacetic acid pH 7.4) containing a protease inhibitor cocktail (Sigma-Aldrich, St. Louis, MI, USA). The homogenates were centrifuged at 14,000× *g* for 30 min at 4 °C. Quantitative evaluation of TNF-α, IL-6, IL-10, IL-1 ra and IL-1β was performed via enzyme-linked immunosorbent assay (DuoSet ELISA, R and D Systems, Minneapolis, MN, USA). Protein concentration was determined using the Bradford assay (Bio-Rad, Hercules, CA, USA).

### 4.9. Insulin Tolerance Test (ITT)

After the last administration of DOX, the animals were subjected to 6 h of fasting, followed by ITT [59]. The caudal capillary glycemia was measured and monitored every 5 min using a glucometer (Accu-Chek Active, Roche, Mannheim, Germany) after the intraperitoneal injection of insulin (0.5 IU/kg body weight) for 30 min. The rate of glucose decay following insulin administration was calculated according to the method described by Bonora et al. (1987) [59].

### 4.10. Glucose Tolerance Test (GTT)

After a 6 h of fasting, the animals received a dose of glucose (2 g/kg body weight) intraperitoneally. The determination of caudal capillary glycemia was determined using a glucometer (Accu-Chek Active, Roche) at different time points: 0, 15, 30, 60 and 90 min after glucose administration.

### 4.11. Gene Expression

Total RNA was extracted from the gastrocnemius muscle, and tumor using the Trizol reagent (Invitrogen Life Technologies, Grand Island, NY, USA) and reverse transcribed to cDNA using the High Capacity cDNA kit (Applied Biosystems, Foster City, CA, USA). Gene expression was determined via real-time PCR using the StepOnePlus real-time PCR system (Applied Biosystems) and SYBR Green Master Mix (Applied Biosystems) as a fluorescent dye. Gene expression was evaluated using the comparative Ct method (Ct = threshold cycle, where the PCR product reaches a detection threshold), with RPL-19 as the constitutive gene. The primer sequences for real-time PCR are listed in Table S1.

### 4.12. Puromycin Assay

Protein synthesis was evaluated according to SUnSET method [60]. Animals received an intraperitoneal dose of puromycin (0.04 μmol/g) dissolved in 200 μL of autoclaved phosphate-buffered saline. After 30 min the gastrocnemius muscle was removed. The levels of puromycin-bound proteins were quantified using Western blotting.

### 4.13. Western Blotting

The gastrocnemius muscle was homogenized in an extraction buffer containing protease and phosphatase inhibitors. After centrifugation, the proteins present were determined using the Bradford assay (Bio-Rad). Aliquots of each sample were then diluted with the same total protein concentration (50 μg) in Laemmli buffer (Bio-Rad, Hercules, CA, USA) and subjected to sodium dodecyl sulfate–polyacrylamide gel electrophoresis, transferred on to a polyvinylidene membrane and then incubated with primary antibodies against Beclin, Atg7, Atg3, Atg5 and NF-κBp65 (Cell Signaling Technology, Danvers, MA, USA); puromycin (EMD Millipore, Billerica, MA, USA); TLR-4 and GAPDH (Santa Cruz Biotechnology, Santa Cruz, CA, USA) and then incubated with an anti-peroxidase conjugated anti-IgG antibody. After incubation, these membranes were further incubated with the peroxidase substrate (ECL Clarity TM, Bio-Rad) and quantified via optical densitometry.

### 4.14. Statistical Analysis

Statistical analysis was performed using the GraphPad Prism version 6.0 software for Windows (San Diego, CA, USA). Data are expressed as mean and mean standard error. For between-group comparisons, the one-way ANOVA was performed, followed by Bonferroni post hoc test or two-way ANOVA, followed by the Bonferroni post hoc test, as appropriate. Differences were considered significant when *p* < 0.05. Survival analyses were performed using the Kaplan–Meier curve and were compared using the log-rank test for trend. Differences were considered significant when *p* < 0.05.

## 5. Conclusions

The combination of EXER and chemotherapy administration did not impair the antitumor effects of this anthracycline, thereby demonstrating its potential as a safe strategy. Moreover, the practice of exercise combined with DOX minimized the resumption of tumor growth and increased survival compared with the group treated with the chemotherapeutic agent alone. Finally, EXER decreased the proteolytic activity one week after the end of chemotherapy, whereas the group treated with DOX exhibited increased muscle catabolism. These results clearly indicate that exercise mitigates some of the side effects of chemotherapy. Beyond the immediate effects during a chemotherapy regimen, exercise can be used during and after chemotherapy as a countermeasure against cancer cachexia, which arises from both the tumor and treatment.

## Supplementary Materials

The following are available online at https://www.mdpi.com/2072-6694/12/11/3466/s1. Table S1: Sequences of the primers used in real time-PCR, Table S2: Effects of only doxorubicin and the combination of doxorubicin plus endurance exercise on the muscle and adipose tissue weight. Table S3: Levels of pro- and anti-inflammatory cytokines in the gastrocnemius muscle after the chemotherapy treatment, Table S4: Serum parameters, Table S5: Glucose metabolism in tumor-bearing mice, Figure S1: Uncropped Western Blots of Figure 4, Figure S2: Uncropped Western Blots of Figure 5, Figure S3: Uncropped Western Blots of Figure 6, Figure S4: Effect of treatments on glucose metabolism immediately after the interruption of chemotherapy.
