# Supplementary material for: Exercise Reduces the Resumption of Tumor Growth and Proteolytic Pathways in the Skeletal Muscle of Mice Following Chemotherapy
Source: Cancers (Basel). 2020 Nov 20;12(11):3466. doi: 10.3390/cancers12113466 (PMC7699885; doi:10.3390/cancers12113466)
Supplement: Supplementary file 1 [file cancers-12-03466-s001.pdf]

Supplementary Material: Exercise Reduces the Resumption of Tumour Growth and Proteolytic Pathways in the Skeletal Muscle of Mice Following Chemotherapy

Edson Alves de Lima, Jr., Alexandre Abilio de Souza Teixeira, Luana Amorim Biondo, Tiego Aparecido Diniz, Loreana Sanches Silveira, Dario Coletti, Sílvia Busquets Rius and José Cesar Rosa Neto

Table S1. Sequences of the primers used in real time-PCR.

Gene	Left Primer	Right Primer	GenBank*
Trim63	5′-GTGTGAGGTGCCTACTTGCTC-3′	5′-GCTCAGTCTTCTGTCCTTGGA-3′	NM_001039048.2
Fbxo32	5′-ACAAAGGAAGTACGAAGGAGCG-3′	5′-GGCAGTCGAGAAGTCCAGTC-3′	NM_026346.3
Foxo1	5′-CCCAGGCCGGAGTTTAACC-3′	5′-GTTGCTCATAAAGTCGGTGCT-3′	NM_019739.3
Foxo3	5′-CTGGGGGAACCTGTCCTATG-3′	5′-TCATTCTGAACGCGCATGAAG-3′	NM_019740.2
Il6	5′-GTTGTGCAATGGCAATTCTG-3′	5′-CCAGTTTGGTAGCATCCATC-3′	NM_001314054.1
Msnt	5′-AGTGGATCTAAATGAGGGAGT-3′	5′-GGAGTACCTCGTGTTTTGTCTC-3′	NM_010834.3
Rpl19	5′-CAATGCCAACTCCCGTCA-3′	5′-GTGTTTTTCCGGCAAACGAG-3′	NM_009078.2

* https://www.ncbi.nlm.nih.gov/nuccore/.

Table S2. Effects of only doxorubicin and the combination of doxorubicin plus endurance exercise on muscle and adipose tissue weight.

Parameters	Experimental Groups					
Tissue Weight	21st day			28th day		
	LLC	LLC + DOX	LLC + DOX + EXER	LLC	LLC + DOX	LLC + DOX + EXER
Tibialis anterior	68.79 ± 2.24	64.67 ± 1.99	69.33 ± 2.09	65.00 ± 3.27	60.30 ± 3.60	71.89 ± 3.74 #
EDL	25.00 ± 3.90 aa	22.11 ± 2.94 a	22.22 ± 1.96 a	16.50 ± 0.56	14.30 ± 0.73	14.89 ± 0.56
Soleus	14.50 ± 0.77	12.47 ± 0.50 *	12.08 ± 0.62 *	13.70 ± 0.33	12.00 ± 0.65	14.11 ± 0.70
Adiposity index	531.79 ± 32.25 aa	311.07 ± 49.08 ***aa	388.58 ± 50.41 *a	338.00 ± 41.90	125.60 ± 12.23 **	208.89 ± 16.50

Values represent the mean and standard error of the data obtained (mg). n = 15–9. * $p < 0.05$; ** $p < 0.01$; *** $p < 0.001$ vs. LLC group; # $p < 0.05$ compared with the LLC + DOX group; a: $p < 0.05$ compared with the respective group within 28 days; aa: $p < 0.01$ compared with the respective group within 28 days. LLC = Lewis lung carcinoma; DOX = doxorubicin; EXER = endurance exercise.

Table S3. Levels of pro- and antiinflammatory cytokines in the gastrocnemius muscle after the chemotherapy treatment.

Parameters	Experimental Groups		
Cytokines	21st day		
	LLC	LLC + DOX	LLC + DOX + EXER
TNF-a	38.48 ± 23.58	59.5 ± 23.08	60.42 ± 16.09
IL-1β	20.97 ± 12.92	10.43 ± 1.40	12.14 ± 2.61
IL-1ra	28.93 ± 8.32	67.29 ± 25.88	76.82 ± 26.16
IL-10	30.07 ± 7.56	38.06 ± 9.70	69.39 ± 32.50

Values expressed as pg/mg protein. Values represent the mean and standard error of mean of the data obtained. *n* = 9–7. LLC = Lewis lung carcinoma; DOX = doxorubicin; EXER = endurance exercise.

Table S4. Serum parameters.

Parameters	Experimental Groups					
Serum	21st day			28th day		
	LLC	LLC + DOX	LLC + DOX + EXER	LLC	LLC + DOX	LLC + DOX + EXER
Total cholesterol (mg/dL)	174.00 ± 19.52	160.90 ± 21.7	132.50 ± 18.53	126.62 ± 17.54	122.22 ± 4.78	120.95 ± 4.54
Triglycerides (mg/dL)	118.80 ± 3.51	110.80 ± 9.76	127.70 ± 15.56	151.86 ± 20.56	114.79 ± 32.69	89.63 ± 13.95
Lactate (mg/dL)	47.87 ± 3.42	55.48 ± 7.81 a	35.37 ± 1.56 #a	46.16 ± 4.15	103.47 ± 7.18 *	88.31 ± 4.49 *

Values represent the mean and standard error of the mean of the data obtained. *n* = 9–6. * $p < 0.05$ vs. LLC group; # $p < 0.05$ compared with the LLC+DOX group. a: $p < 0.05$ in compared with the respective group on the 28th day. LLC = Lewis lung carcinoma; DOX = doxorubicin; EXER = endurance exercise.

Table S5. Glucose metabolism in tumour-bearing mice.

Parameters	Experimental Groups					
Glucose metabolism	21st day			28th day		
	LLC	LLC + DOX	LLC + DOX + EXER	LLC	LLC + DOX	LLC + DOX + EXER
Glucose (mg/dL)	142.63 ± 10.20 aa	116.7 ± 7.34	117.89 ± 6.55 aa	190.26 ± 22.35	150 ± 10.27	175.40 ± 5.72
Insulin (ng/mL)	1.14 ± 0.51	0.89 ± 0.10	0.92 ± 0.19	1.16 ± 0.14	1.00 ± 0.47	0.87 ± 0.16

Values represent the mean and standard error of the mean of the data obtained. N = 8. aa: $p < 0.01$ compared with the respective group on the 28th day. LLC = Lewis lung carcinoma; DOX = doxorubicin; EXER = endurance exercise.

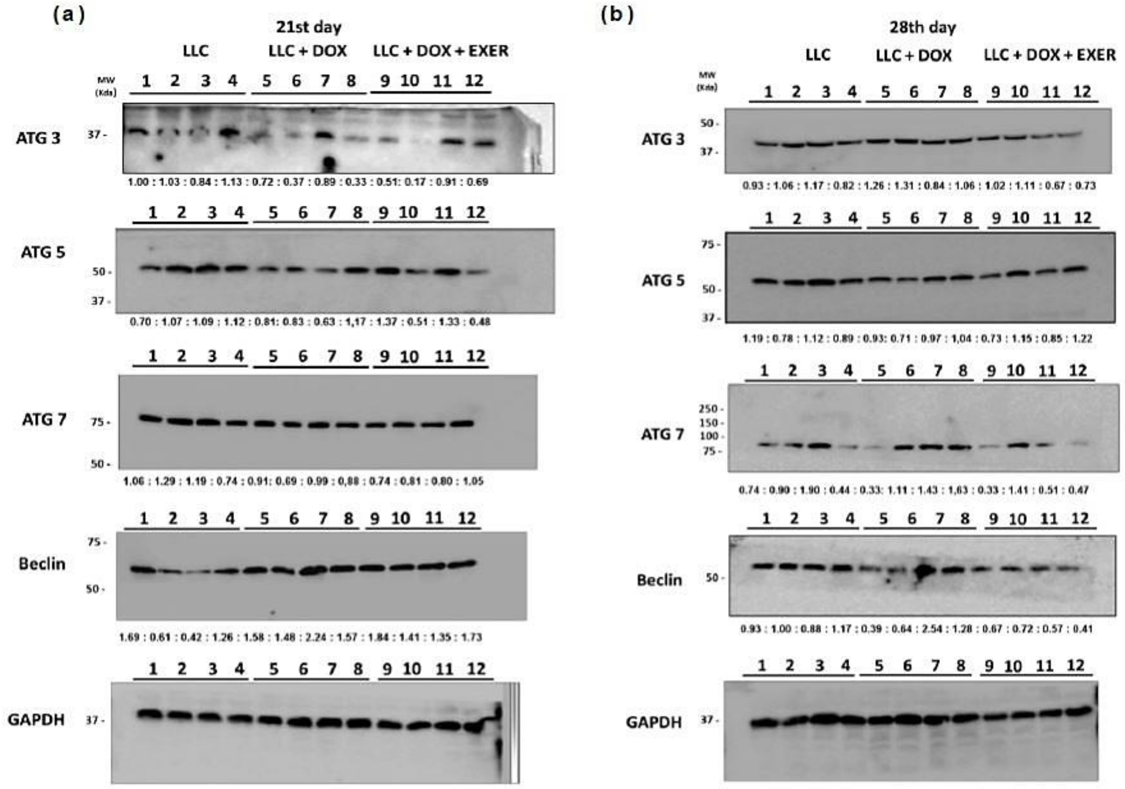

Figure S1. Uncropped Western Blot of Figure 4**.** Autophagic signaling in gastrocnemius. *n* = 4. LLC = Lewis lung carcinoma; DOX = doxorubicin; EXER = endurance exercise.

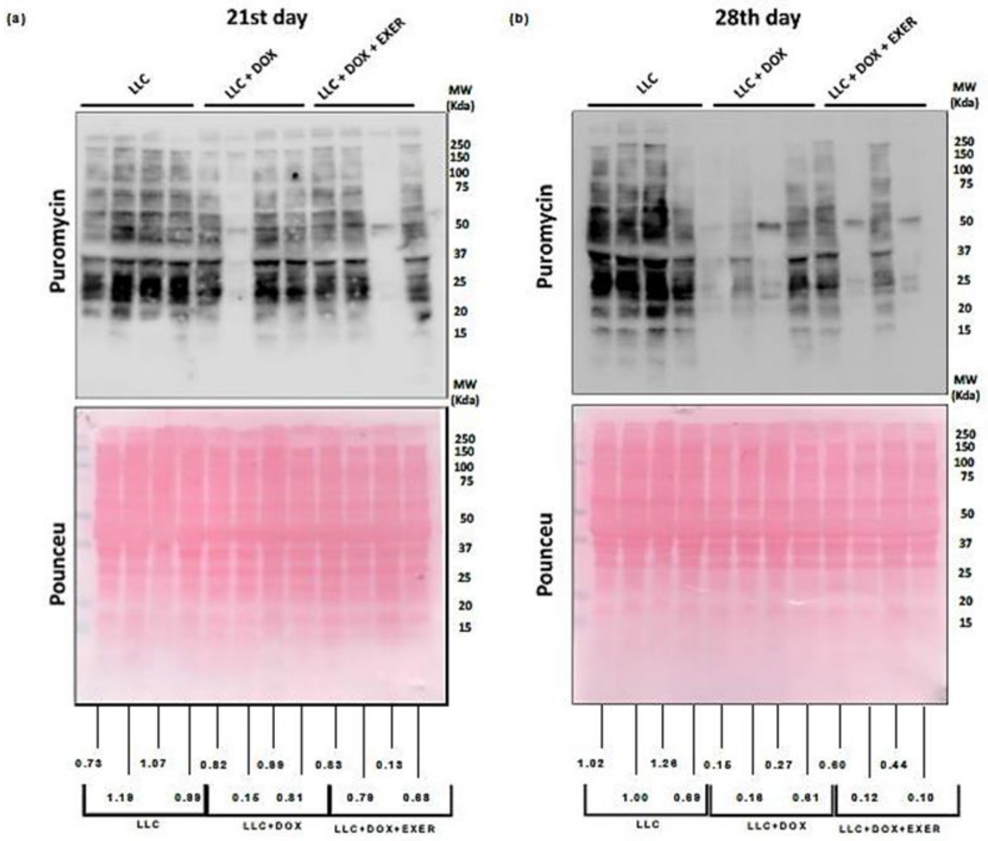

Figure S2. Uncropped Western Blot of Figure 5. Evaluation of total protein synthesis immediately and after chemotherapy. n = 4. LLC = Lewis lung carcinoma; DOX = doxorubicin; EXER = endurance exercise.

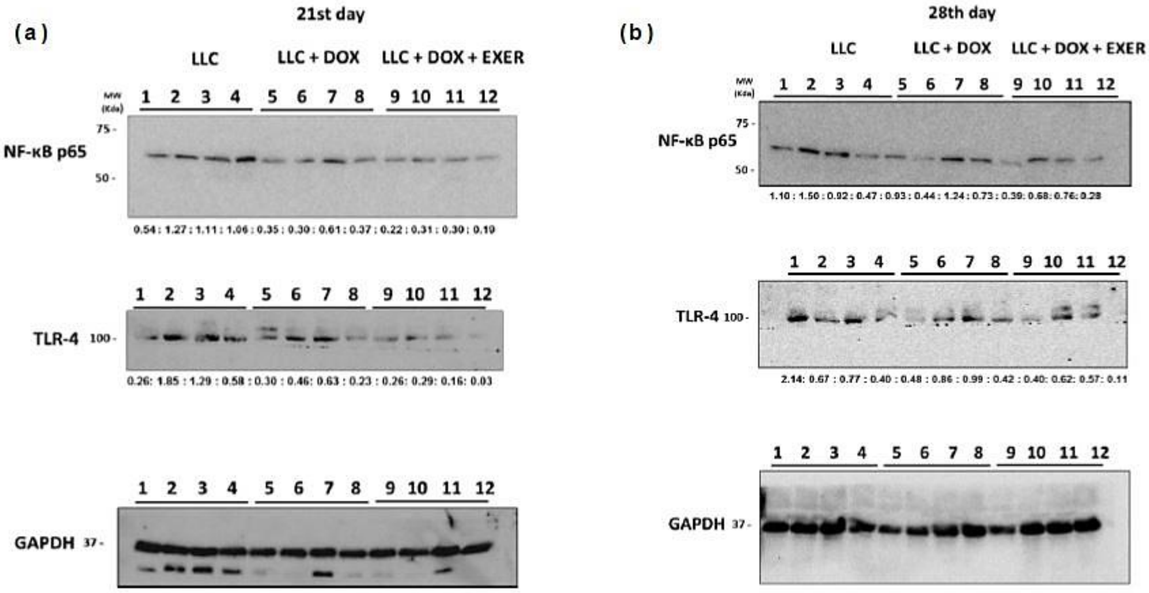

Figure S3. Uncropped Western Blot of Figure 6. Inflammatory signalling in skeletal muscle. n = 4. LLC = Lewis lung carcinoma; DOX = doxorubicin; EXER = endurance exercise.

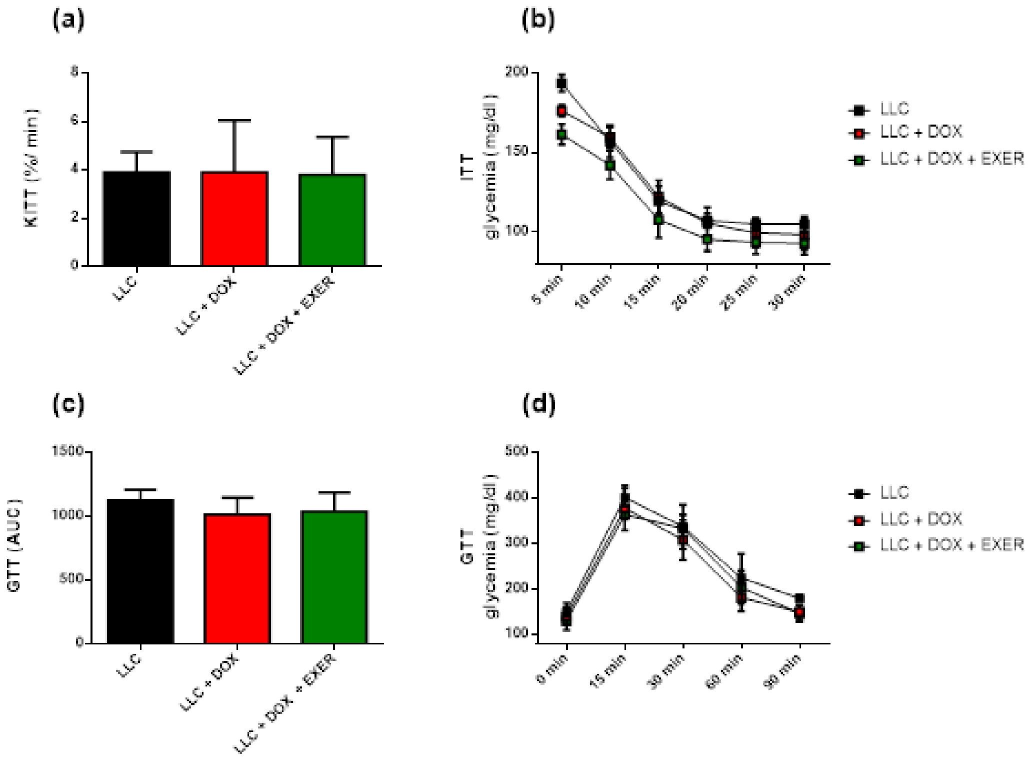

Figure S4. Effect of treatments on glucose metabolism immediately after the interruption of chemotherapy. (**a**). Serum glucose disappearance rate (KiTT) during the insulin tolerance test. n = 14–13; (**b**) Serum glucose levels after intraperitoneal insulin administration (0.5 IU); (**c**) Area under the curve of the glucose tolerance test. n = 5; (**d**) Serum glucose levels after intraperitoneal glucose administration (2 g/kg body weight). Values represent the mean and standard error of the mean of

the analysis of the data obtained. LLC = Lewis lung carcinoma; DOX = doxorubicin; EXER = endurance exercise.

Publisher's Note: MDPI stays neutral with regard to jurisdictional claims in published maps and institutional affiliations.

© 2020 by the authors. Licensee MDPI, Basel, Switzerland. This article is an open access article distributed under the terms and conditions of the Creative Commons Attribution (CC BY) license (http://creativecommons.org/licenses/by/4.0/).
